# Supplementary material for: Reversal of ATP synthase is a key attribute accompanying cellular differentiation of Trypanosoma brucei insect forms
Source: Commun Biol. 2026 Mar 27;9:680. doi: 10.1038/s42003-026-09933-z (PMC13187271; doi:10.1038/s42003-026-09933-z)
Supplement: Supplementary file 1 — Supplementary Information [file 42003_2026_9933_MOESM1_ESM.pdf]

Supplementary Information

for

Reversal of ATP synthase is a key attribute accompanying cellular differentiation of *Trypanosoma brucei* insect forms

Kunzová Michaela[1,2], Doleželová Eva[1], Moos Martin[3], Panicucci Brian[1], Zíková Alena[1,2,*]

[1]Institute of Parasitology, Biology Centre, Czech Academy of Sciences, Ceske Budejovice, Czech Republic

[2]Faculty of Science, University of South Bohemia, Ceske Budejovice, Czech Republic

[3]Institute of Entomology, Biology Centre, Czech Academy of Sciences, Ceske Budejovice, Czech Republic

*Corresponding author: azikova@paru.cas.cz

Supplementary Figures:

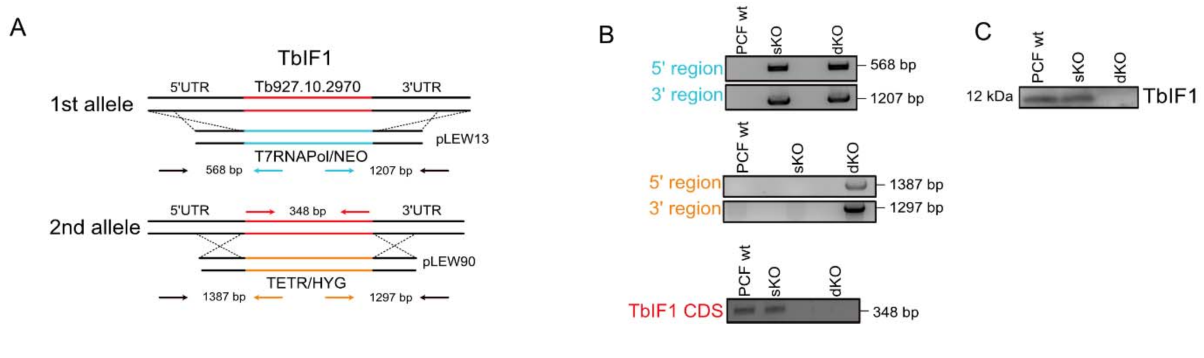

Supplementary Figure 1. Generation of TbIF1 double knock-out (dKO). (A) Scheme of the dKO generation followed by incorporation of RBP6^{OE} cassette to the rRNA spacer. (B) PCR validation of the correct TbIF1 gene allele replacement with pLEW13 and pLEW90 cassettes. (C) Western blot analysis using anti-TbIF1 specific antibody validating the absence of TbIF1 protein in the RBP6OE_TbIF1dKO cells.

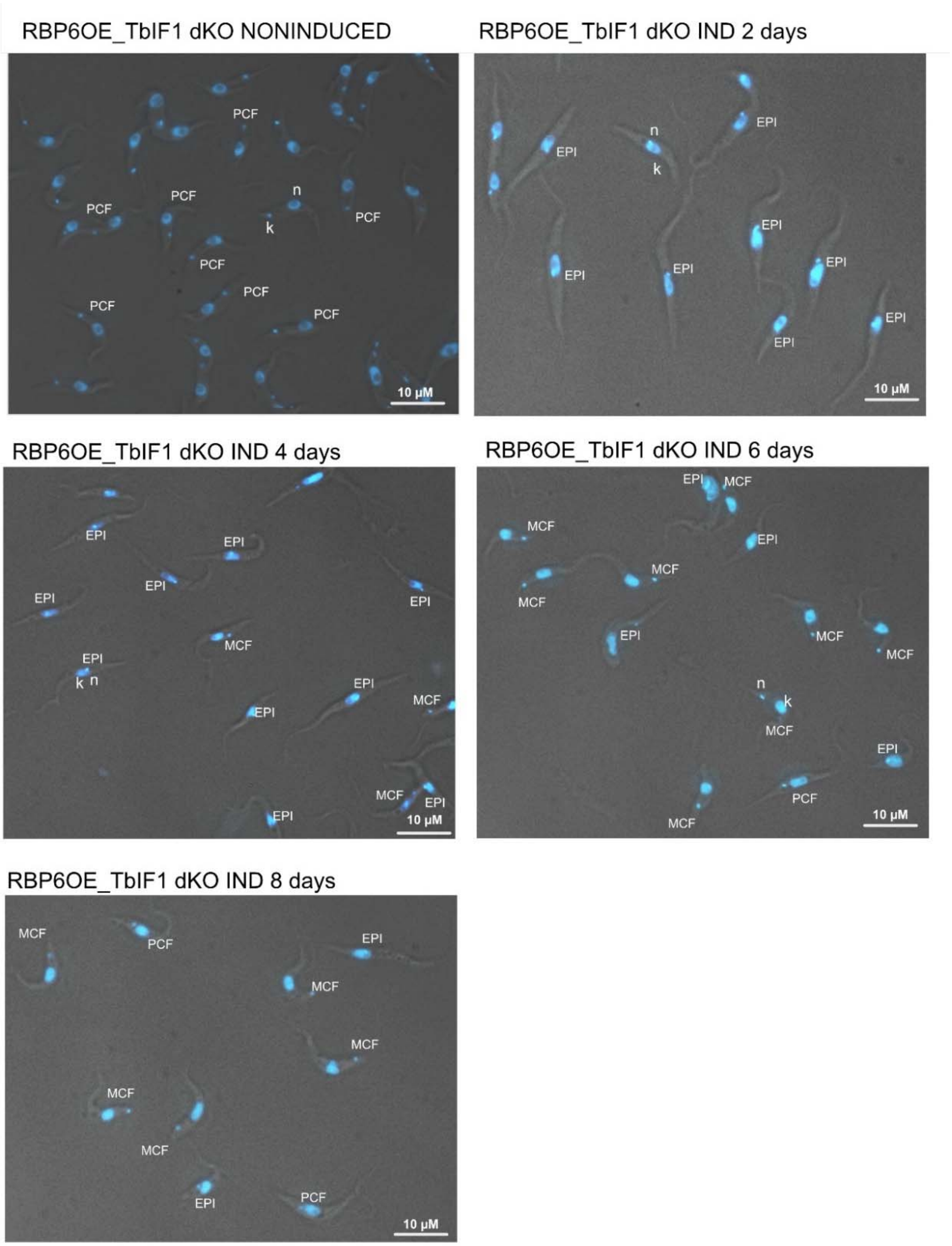

Supplementary Figure 2. Morphological progression of *T. brucei* induced by RBP6 overexpression. Morphological stages of *T. brucei* during RBP6 overexpression. Representative images show differentiation from procyclic forms (PCF) to epimastigote (EPI) and metacyclic forms (MCF) over an 8-day induction period. Samples include non-induced and induced $RBP6^{OE}$_TbIF1 dKO parasites at 2, 4, 6, and 8 days. Panels are labeled to indicate the observed morphological forms.

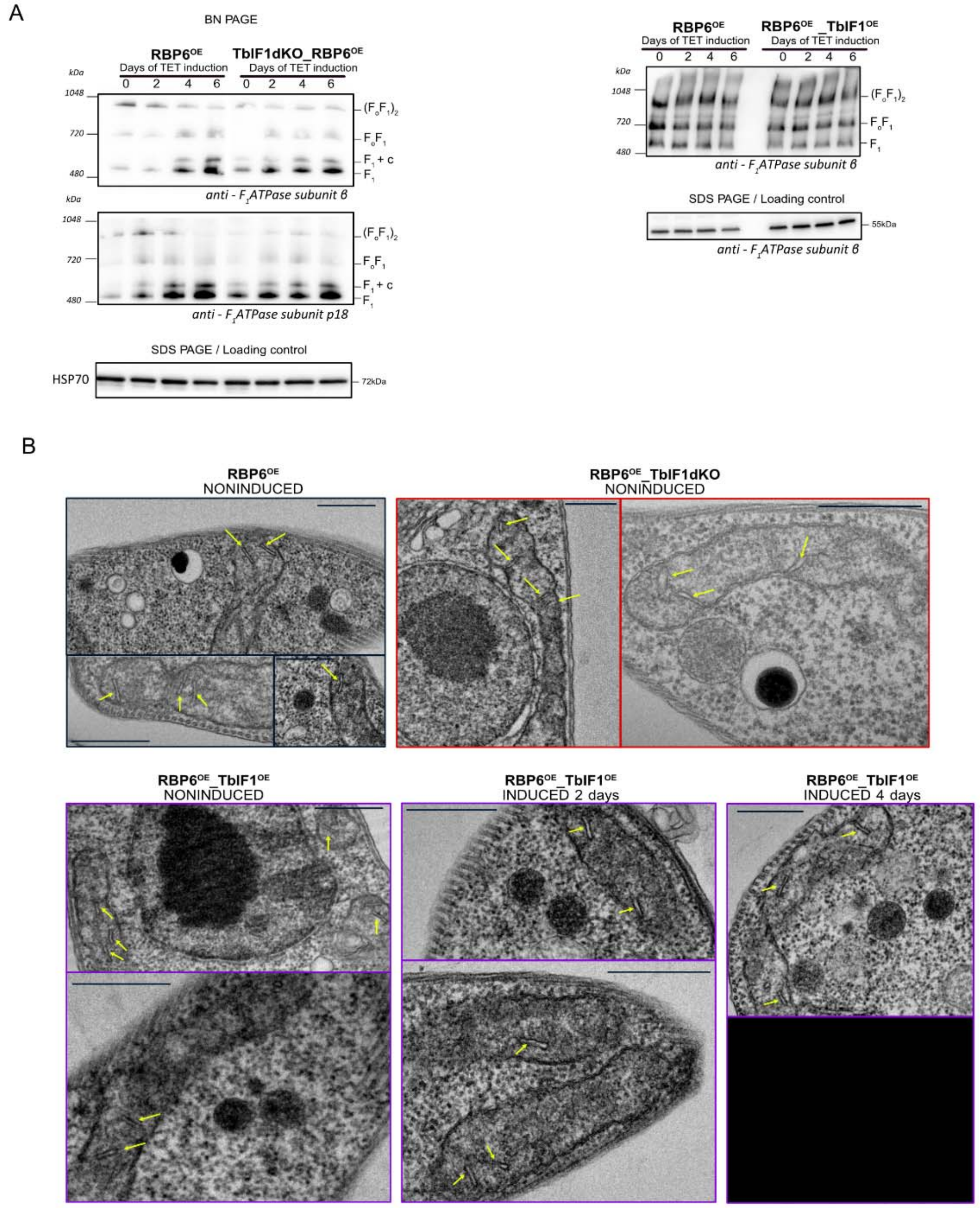

Supplementary Figure 3. Modulation of TbIF1 expression has no effect on the steady state of the ATP synthase monomer and dimer. (A) A western blot analysis of mitochondrial lysates resolved under native conditions using anti-subunit β antibody recognizing the F1 moiety, as well as the monomeric and dimeric ATP synthase complex. Positions of the native marker (NativeMarK Unstained Protein Standard, Invitrogen) are indicated. Mitochondrial lysates were also analyzed by SDS PAGE and western blot analysis using mtHSP70 or anti-subunit β antibody as a loading control. (B) Representative transmission electron micrographs of sections of RBP6^{OE} and RBP6^{OE}_TbIF1dKO noninduced cells as well as RBP6^{OE}_TbIF1^{OE} cells induced for 0, 2 and 4 days. Mitochondrial cristae are marked with yellow arrows. Scale bar 500 nm.

Supplementary Figure 4

Uncropped western blots shown in figures 1A, 1D, 2A, 2C, 5C, 6C and Supplementary Figures S1 and S3

Uncropped agarose gels for Supplementary Figure 1

Figure 1A

RBP6^{OE} cell line

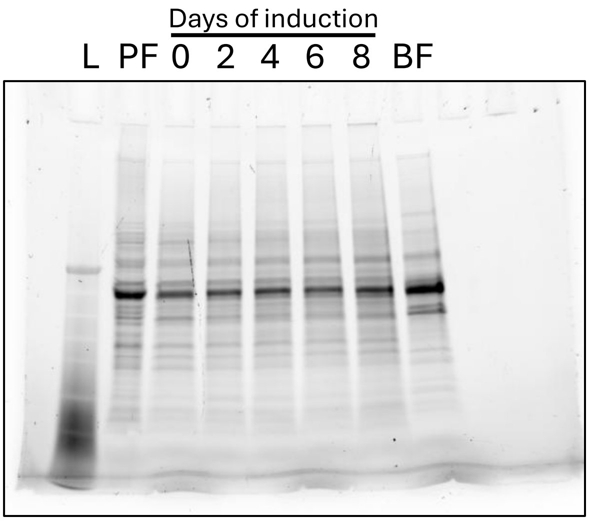

Stain free

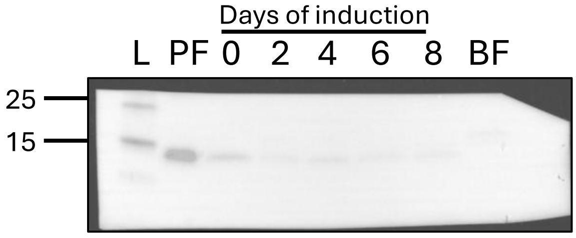

anti-TbIF1

RBP6^{OE} cell line

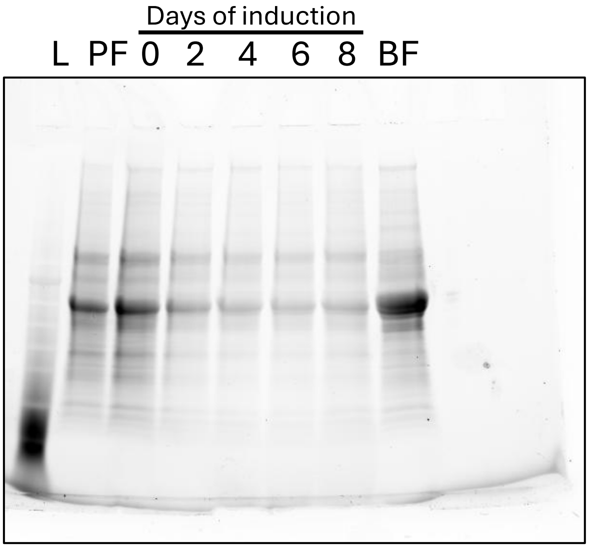

Stain free

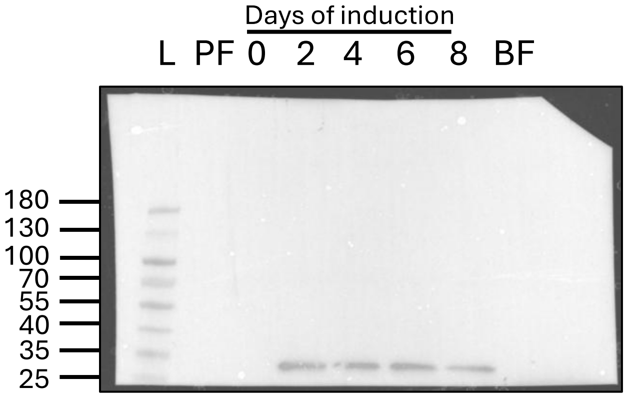

anti-RBP6

TbIF1dKO_RBP6^{OE} cell line

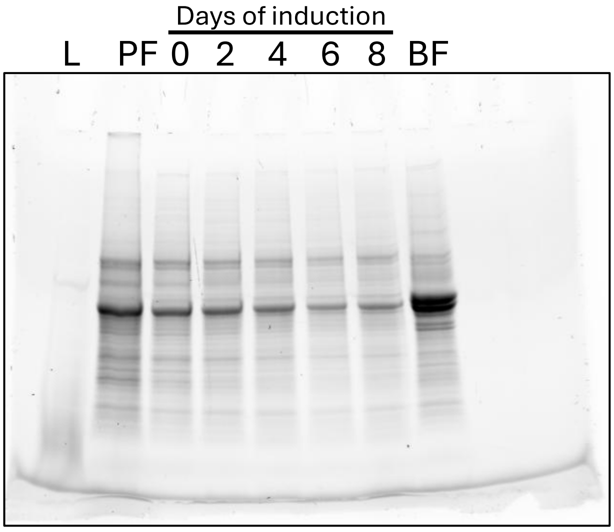

Stain free

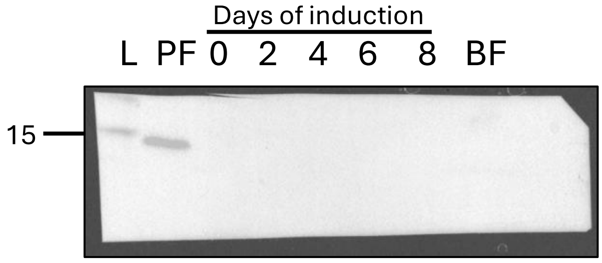

anti-TbIF1

L – ladder
PF – procyclic form wt
BF – bloodstream form wt

Figure 1A

TbIF1dKO_RBP6^{OE} cell line

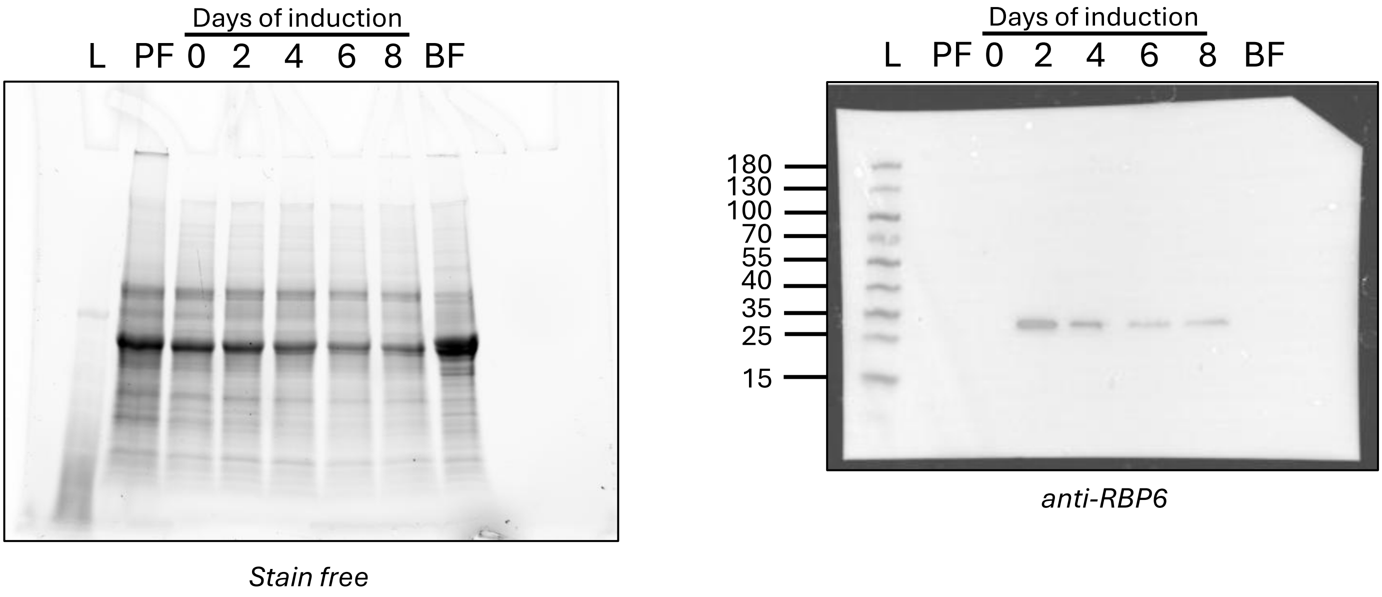

RBP6^{OE}_TbIF1^{OE} cell line

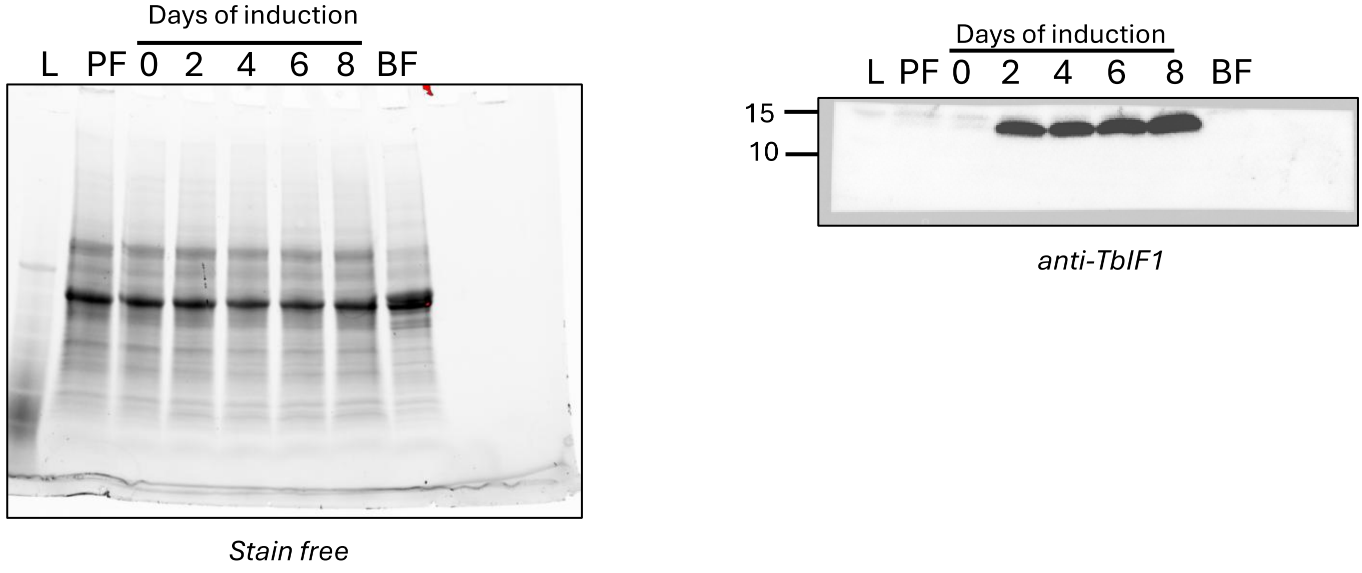

RBP6^{OE}_TbIF1^{OE} cell line

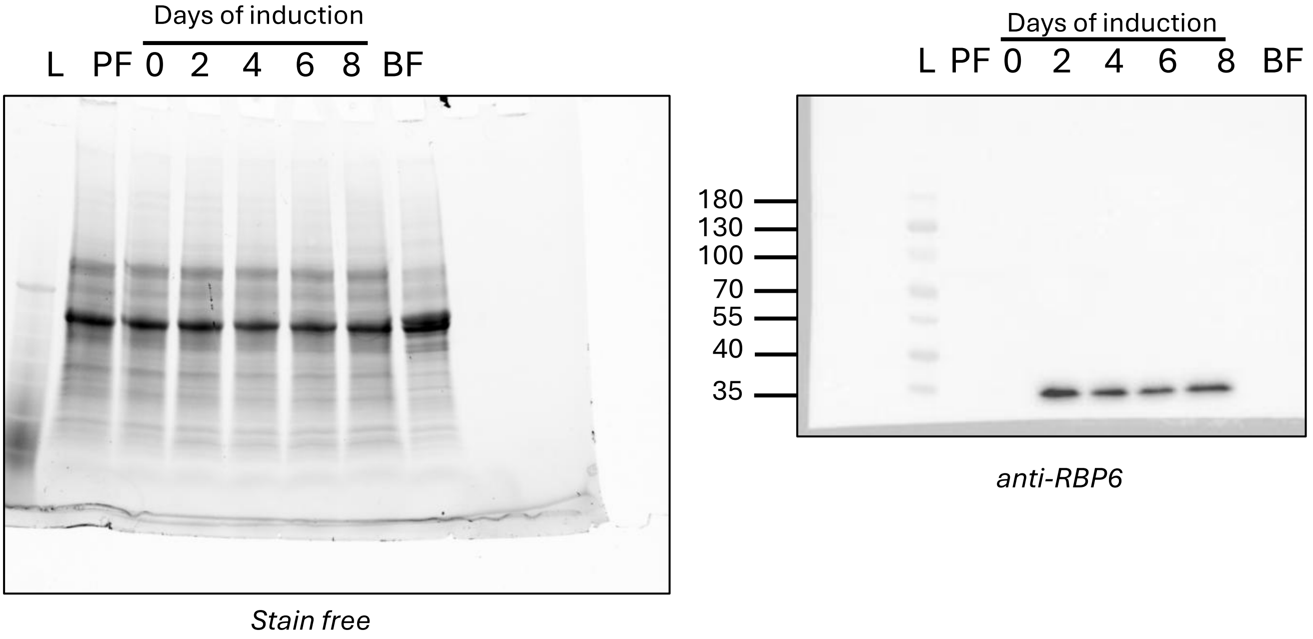

L – ladder
PF – procyclic form wt
BF – bloodstream form wt

Figure 1D

RBP6^{OE} cell line

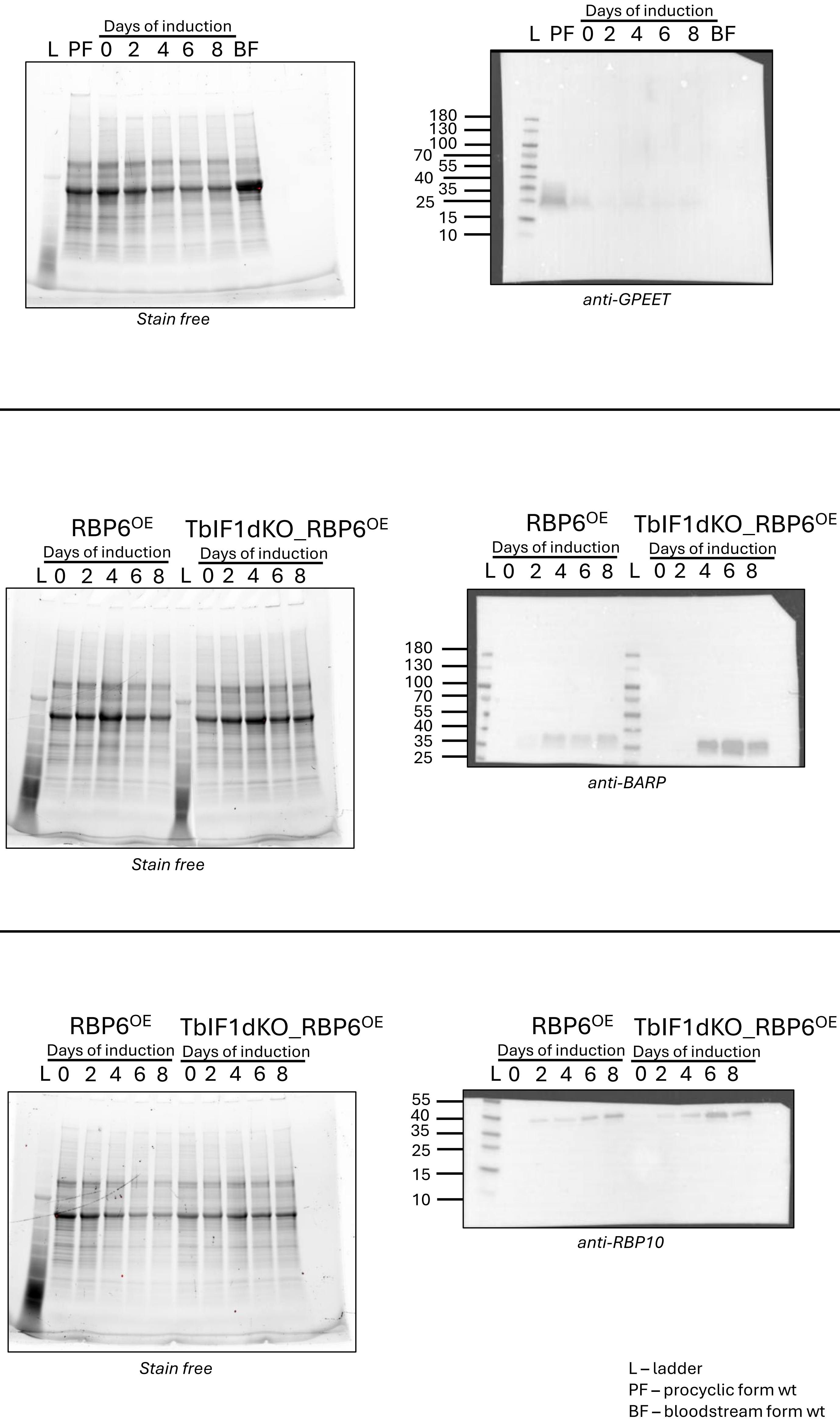

Figure 1D

RBP6^{OE} cell line

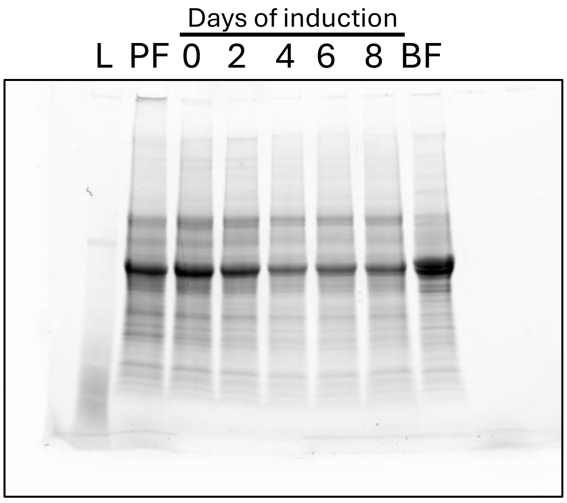

Stain free

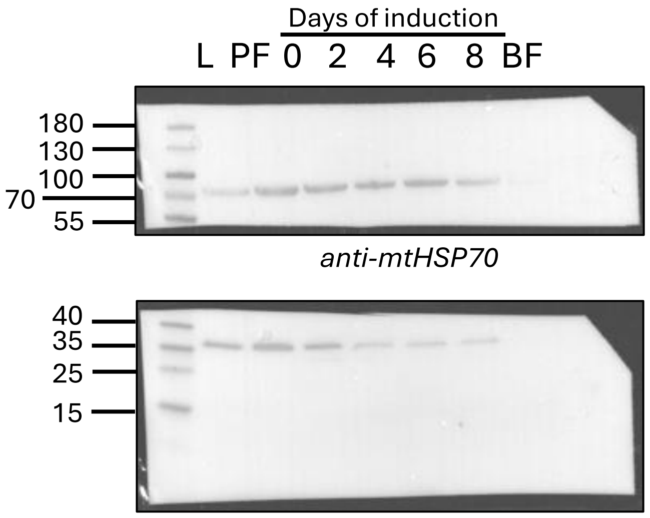

anti-Rieske

RBP6^{OE} cell line

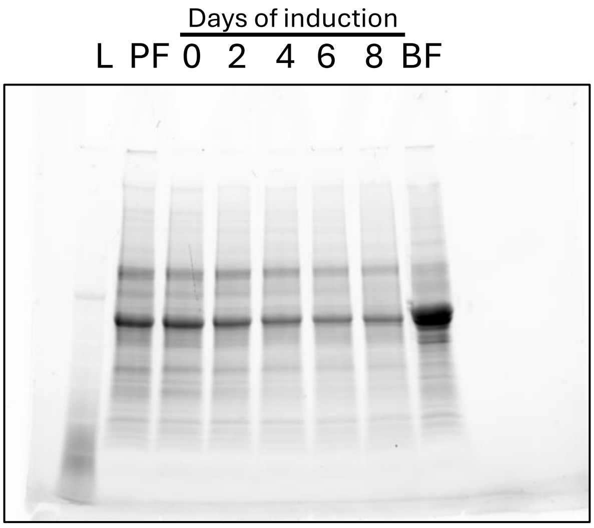

Stain free

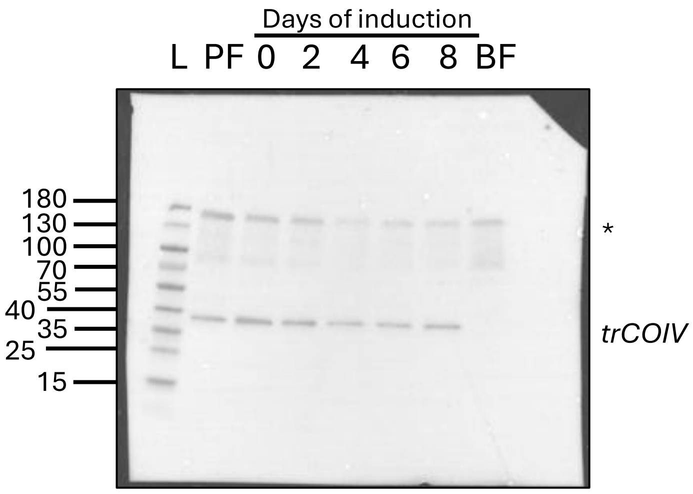

anti-trCOIV

RBP6^{OE} cell line

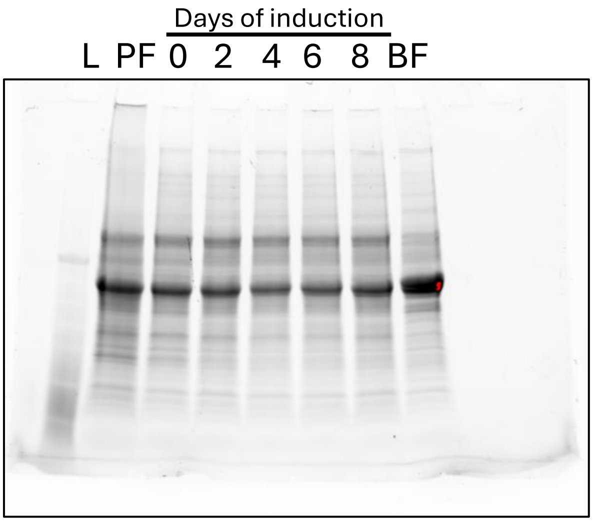

Stain free

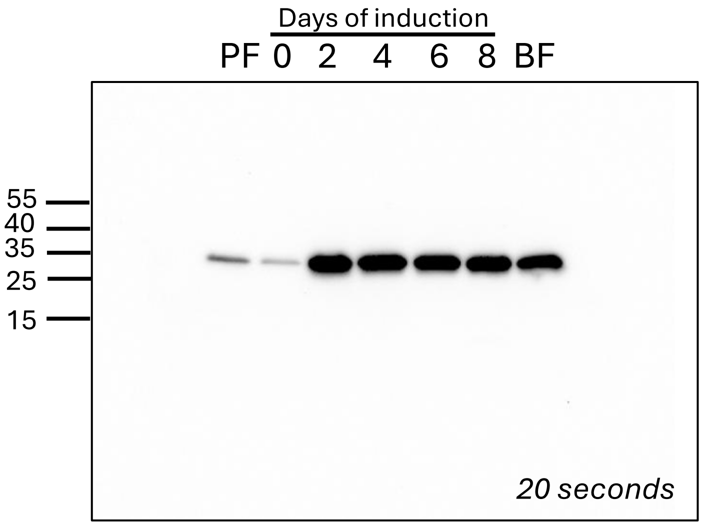

anti-AOX

* - nonspecific band
L – ladder
PF – procyclic form wt
BF – bloodstream form wt

Figure 1D

RBP6^{OE} cell line

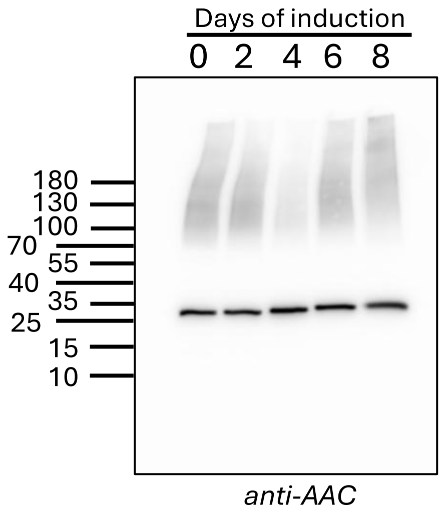

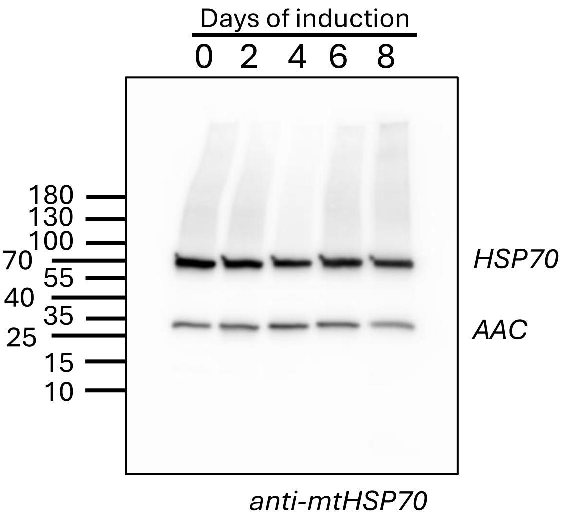

Figure 1D

TbIF1dKO_RBP6^{OE} cell line

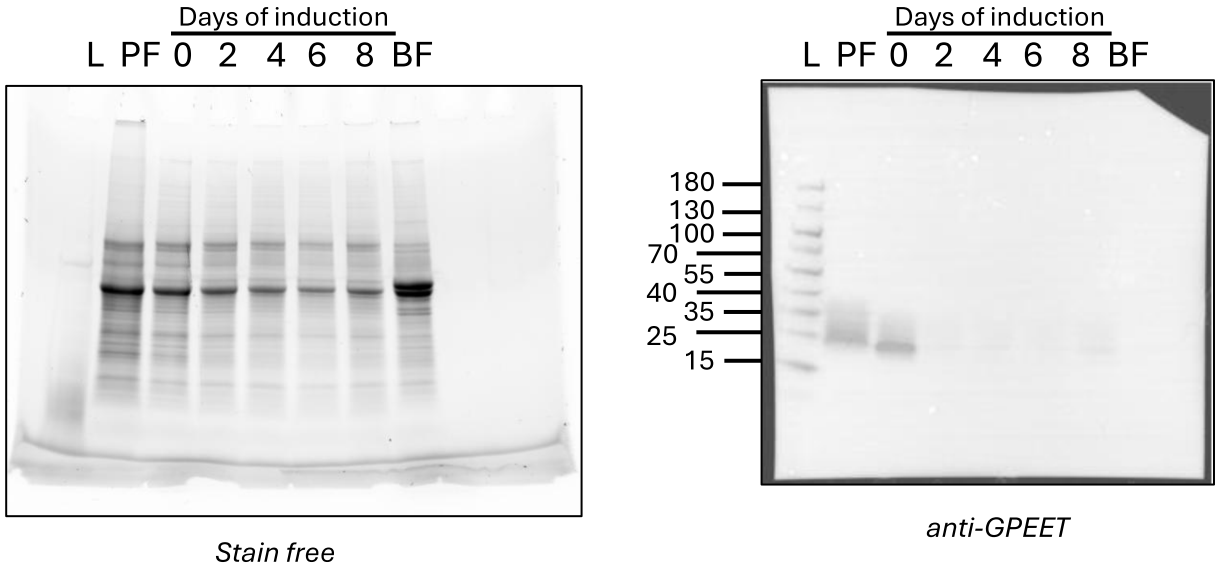

TbIF1dKO_RBP6^{OE} cell line

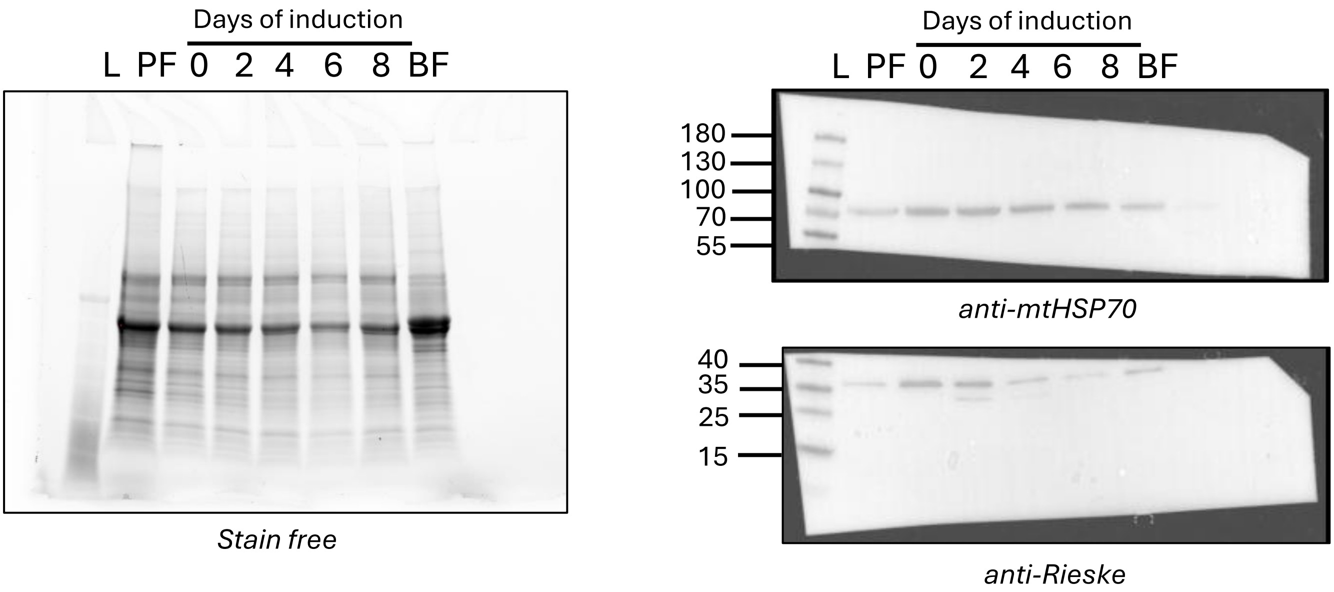

TbIF1dKO_RBP6^{OE} cell line

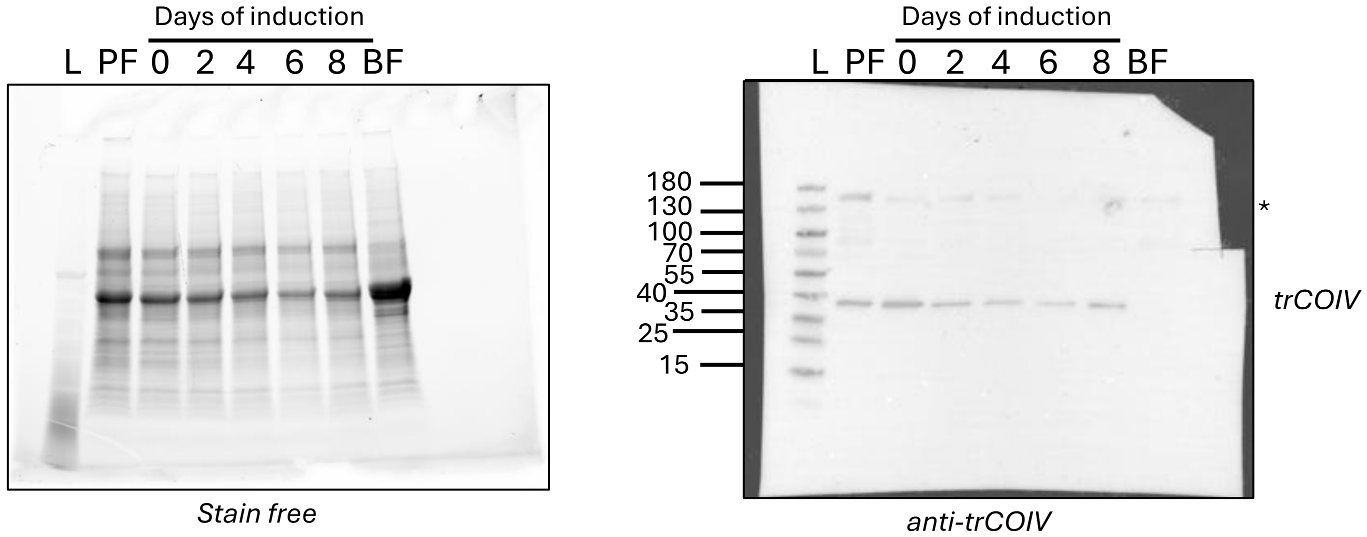

* - nonspecific band
L – ladder
PF – procyclic form wt; BF – bloodstream form wt

Figure 1D

TbIF1dKO_RBP6^{OE} cell line

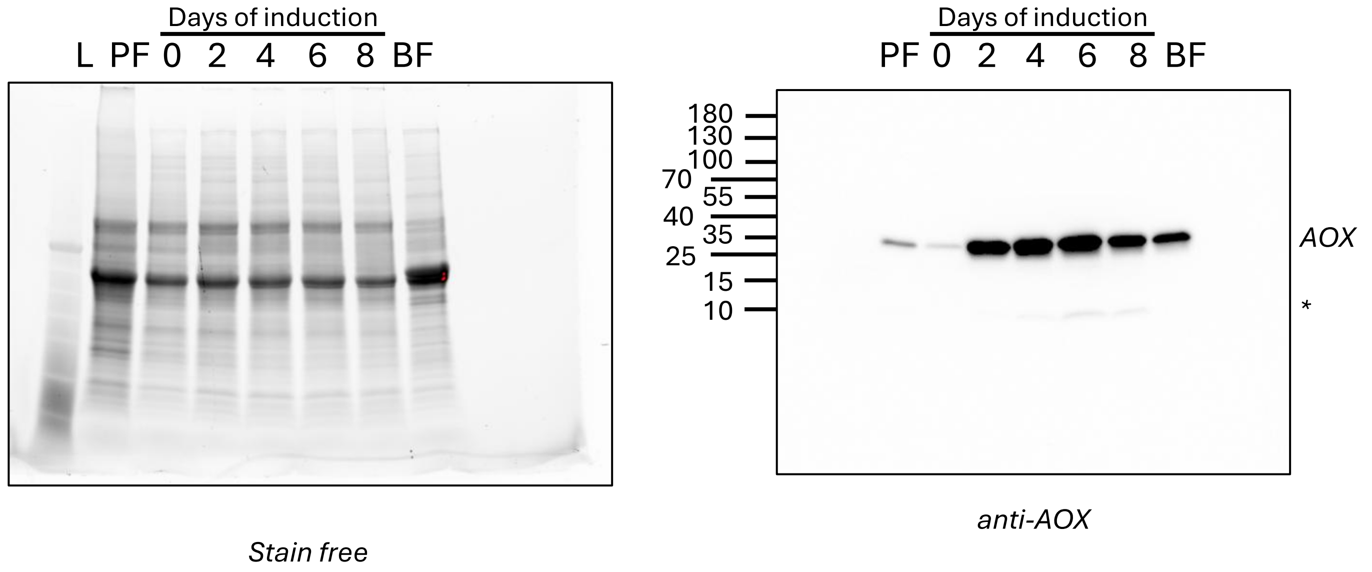

TbIF1dKO_RBP6^{OE} cell line

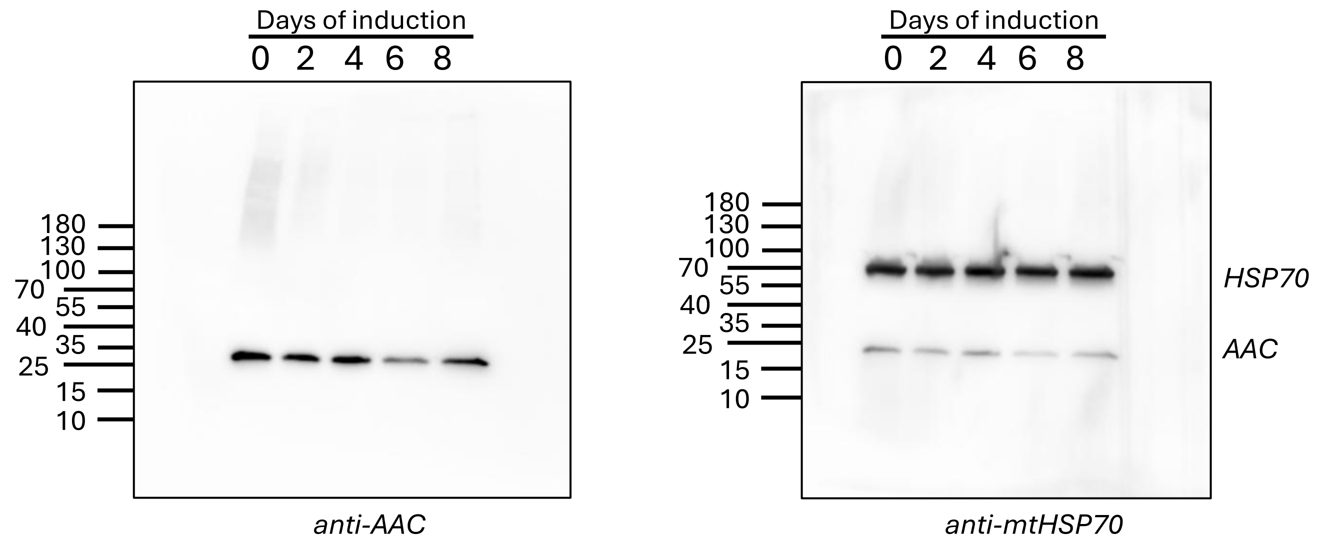

* - nonspecific band
L – ladder
PF – procyclic form wt
BF – bloodstream form wt

Figure 1D

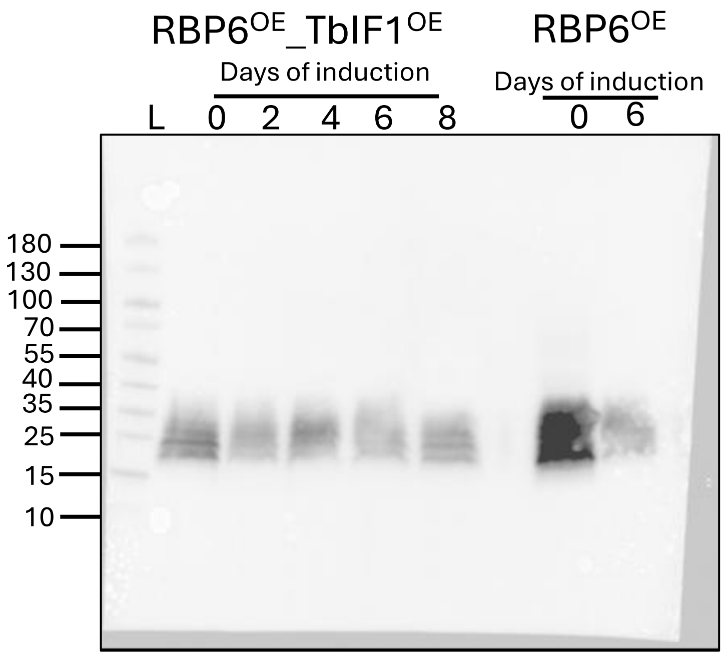

anti-GPEET

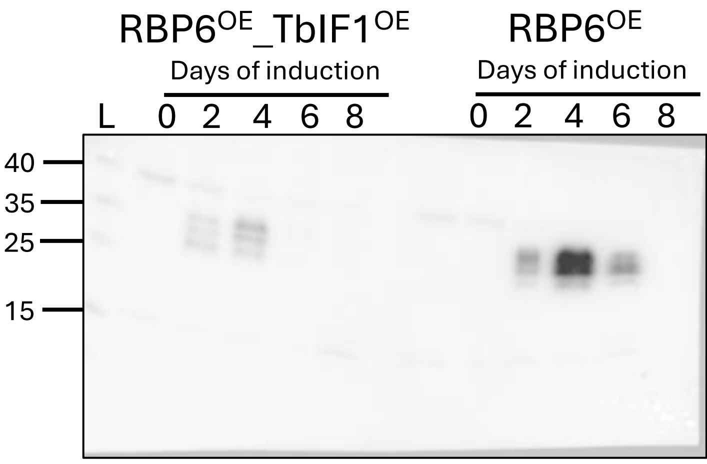

anti-BARP

RBP6^{OE}_TbIF1^{OE} cell line

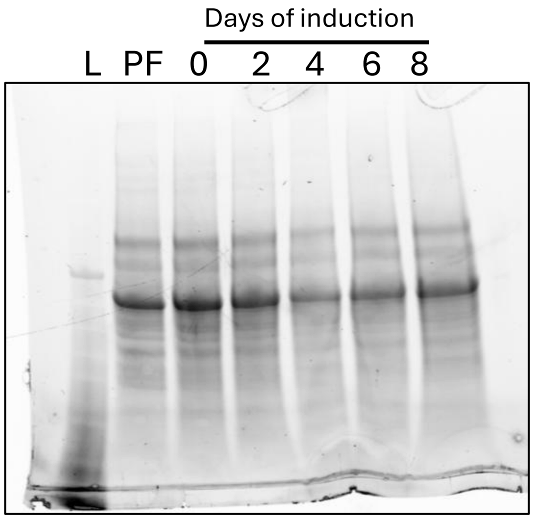

Stain free

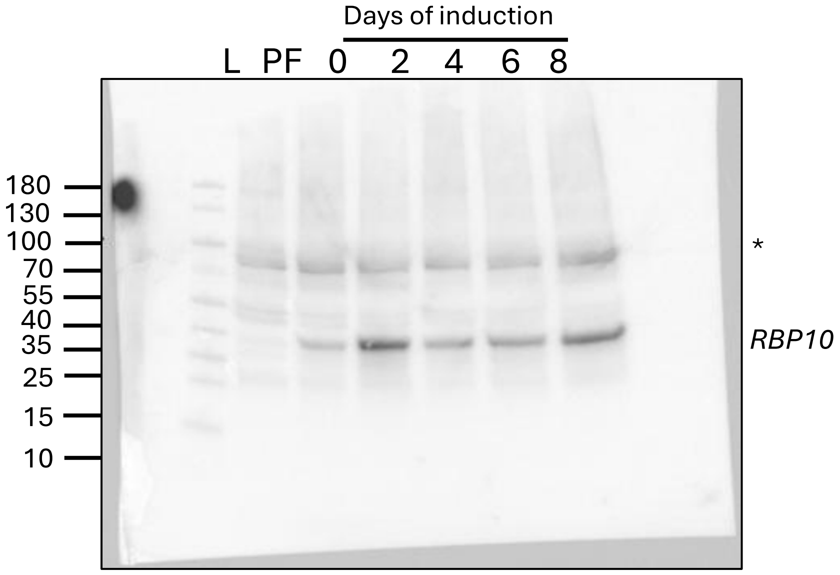

anti-RBP10

* - nonspecific band
L – ladder
PF – procyclic form wt
BF – bloodstream form wt

Figure 1D

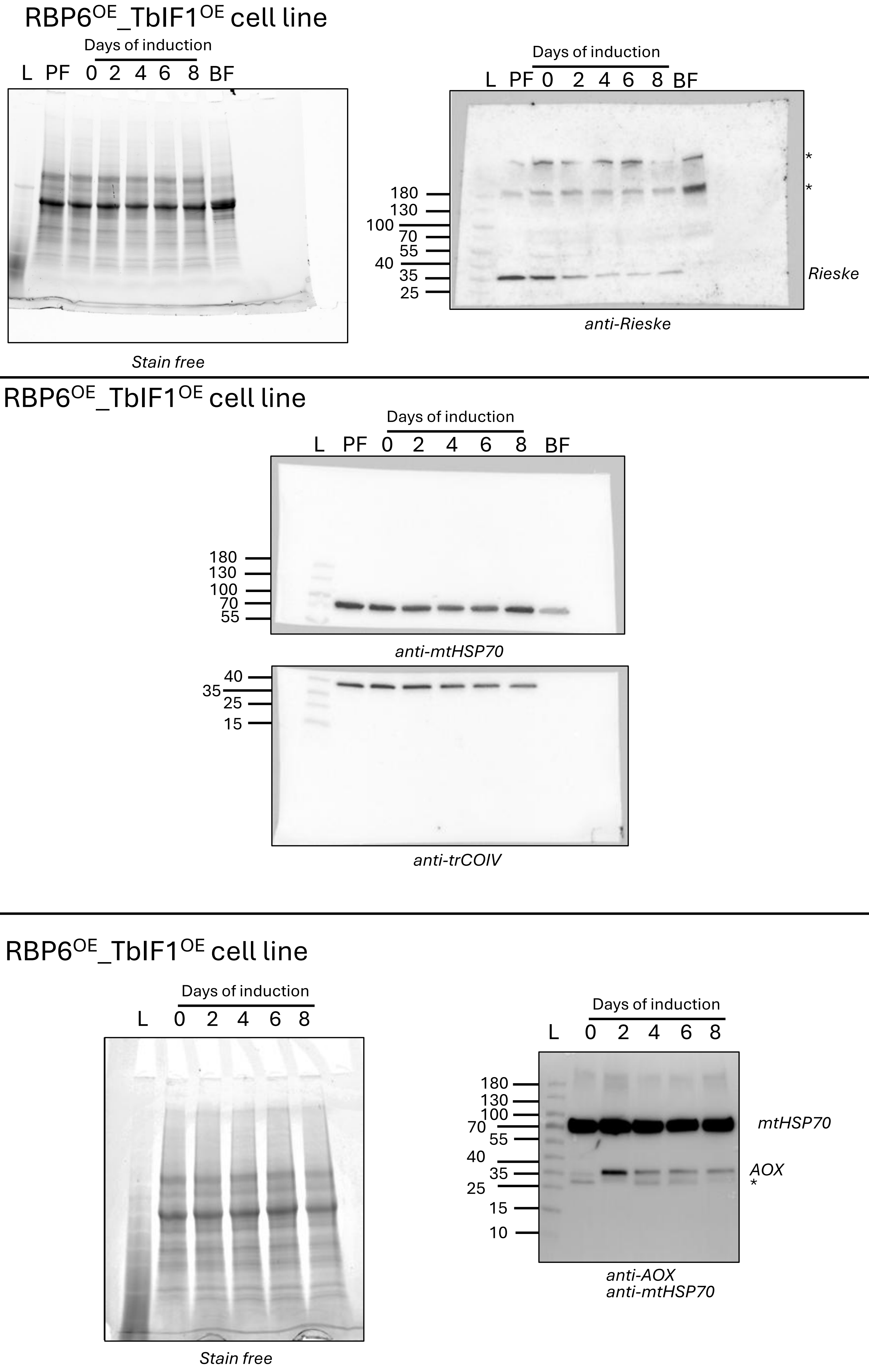

* - nonspecific band
L – ladder
PF – procyclic form wt
BF – bloodstream form wt

Figure 1D

RBP6^{OE}_TbIF1^{OE} cell line

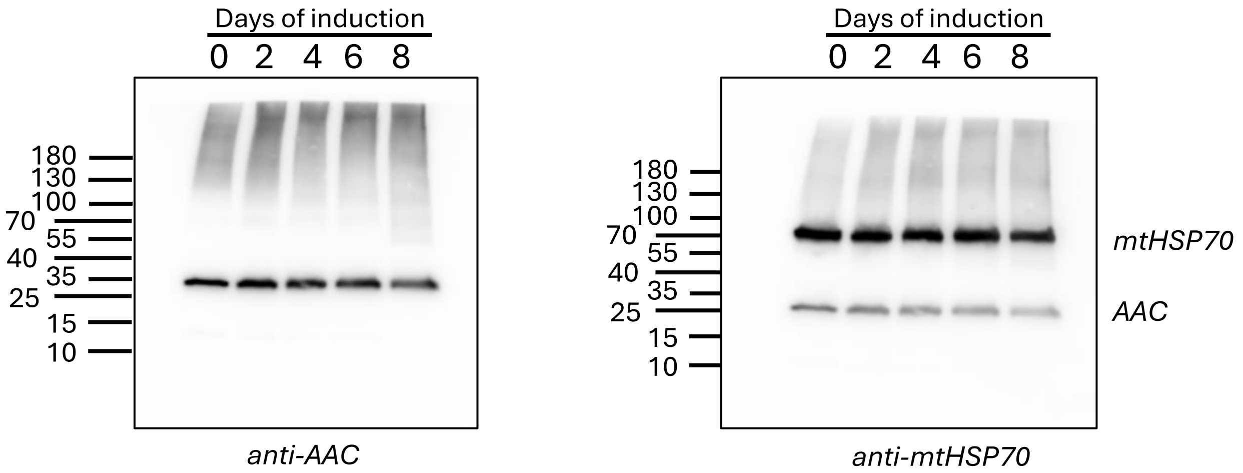

Figure 2A

RBP6^{OE} cell line

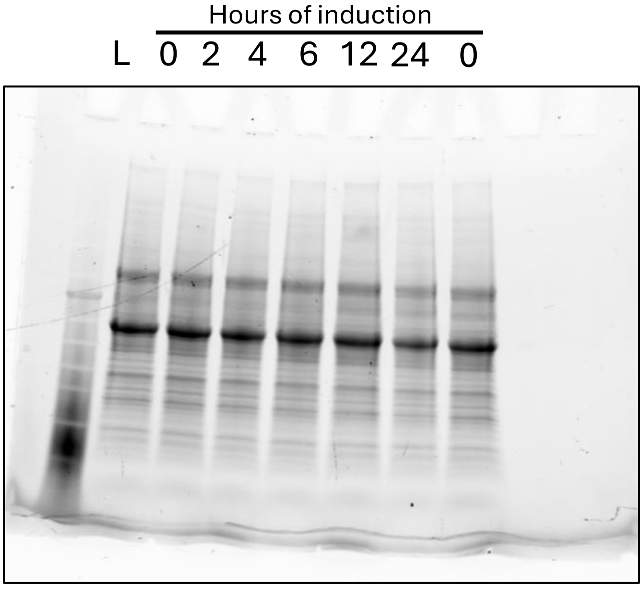

Stain free

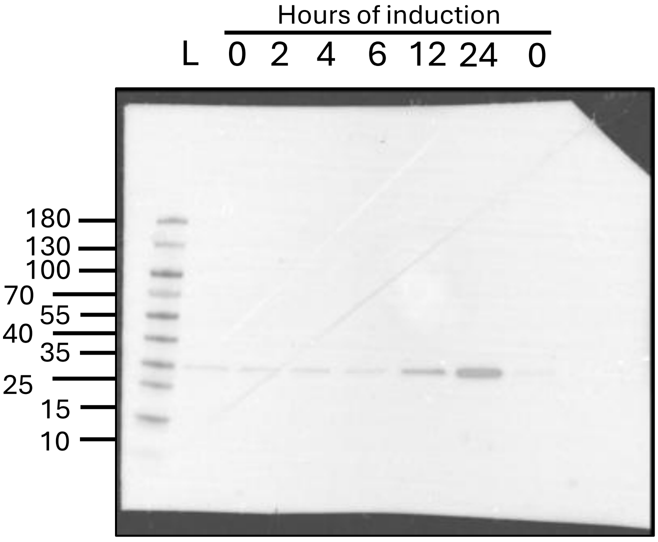

anti-AOX

RBP6^{OE} cell line

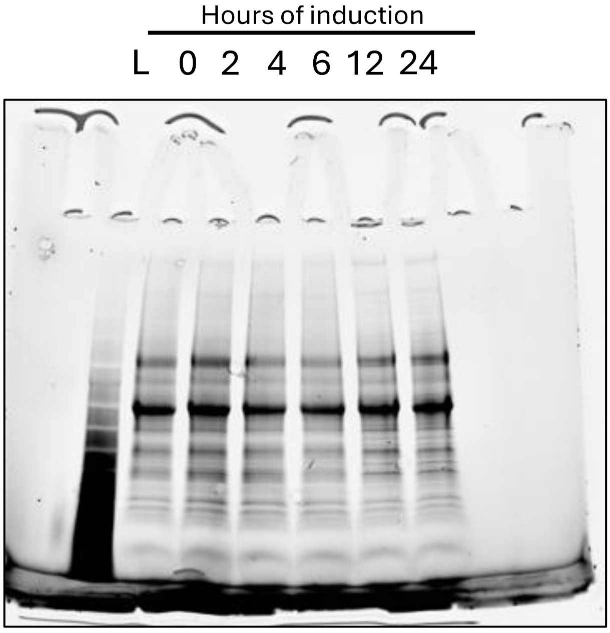

Stain free

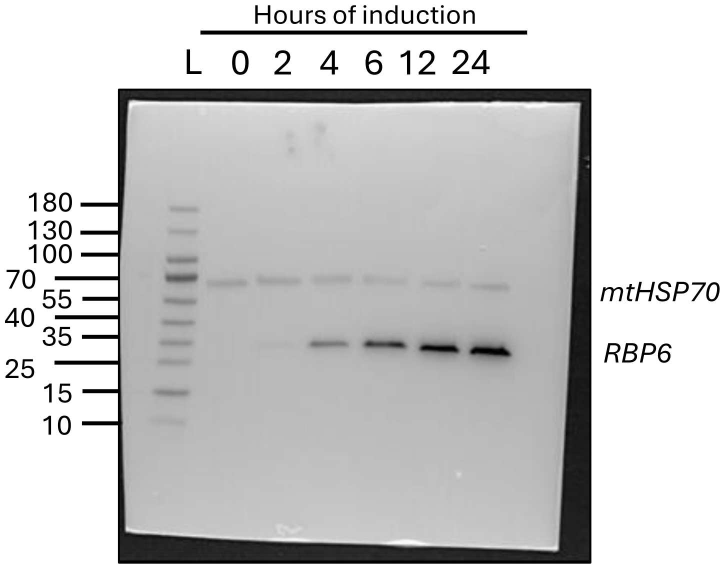

anti-mtHSP70
anti-RBP6

* - nonspecific band
L – ladder
PF – procyclic form wt
BF – bloodstream form wt

Figure 2C

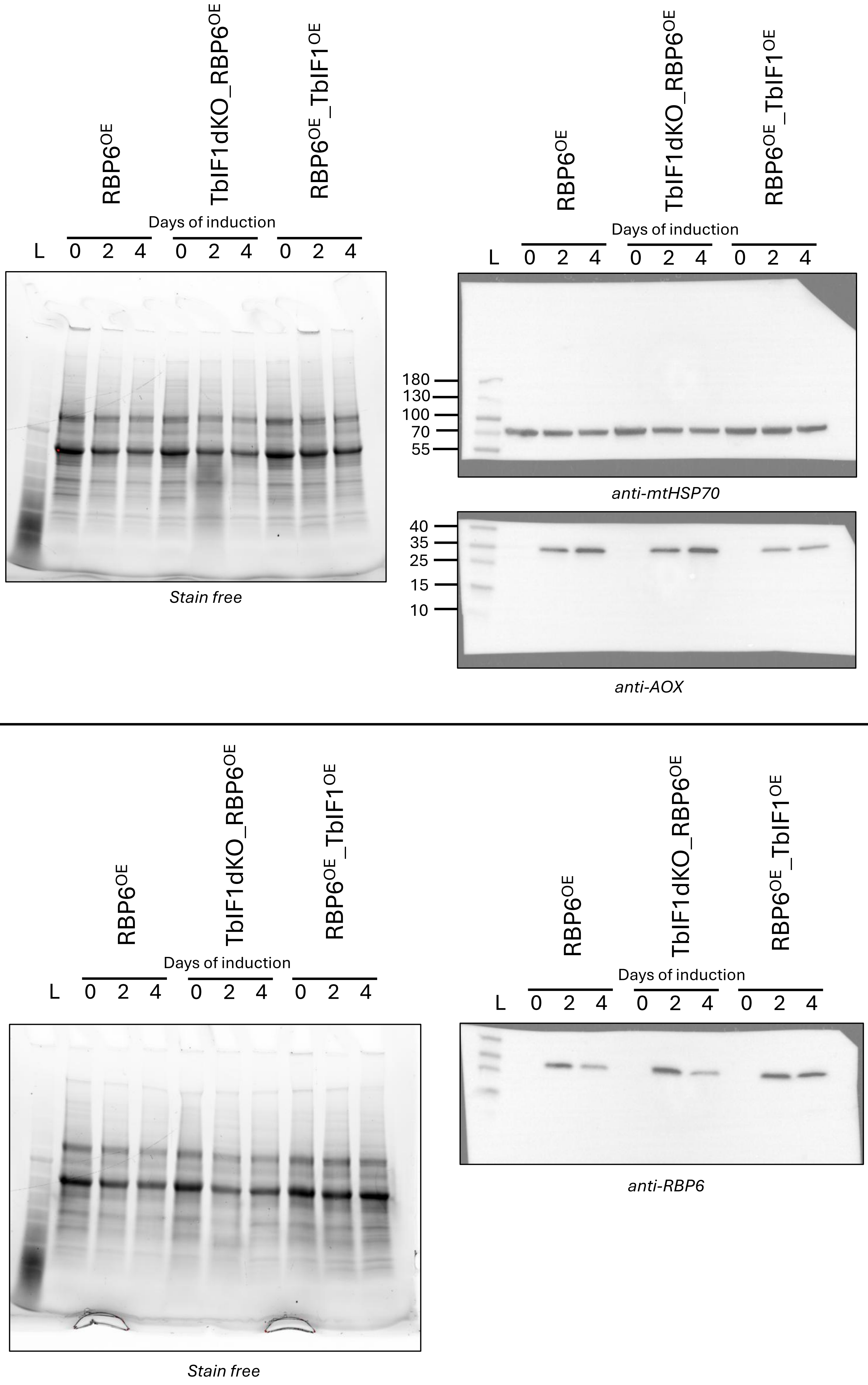

* - nonspecific band
L – ladder

Figure 5C

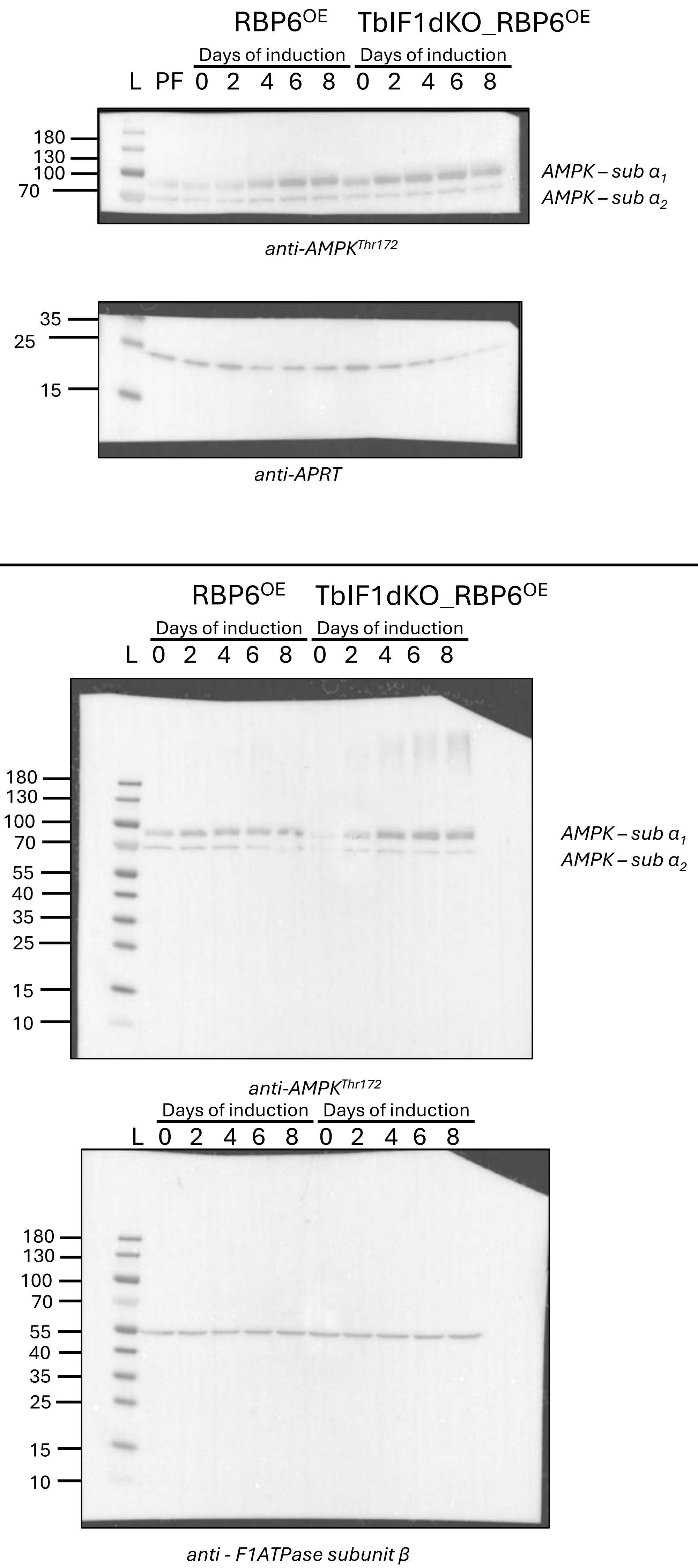

* - nonspecific band

L – ladder

PF – procyclic form wt

BF – bloodstream form wt

Figure 5C

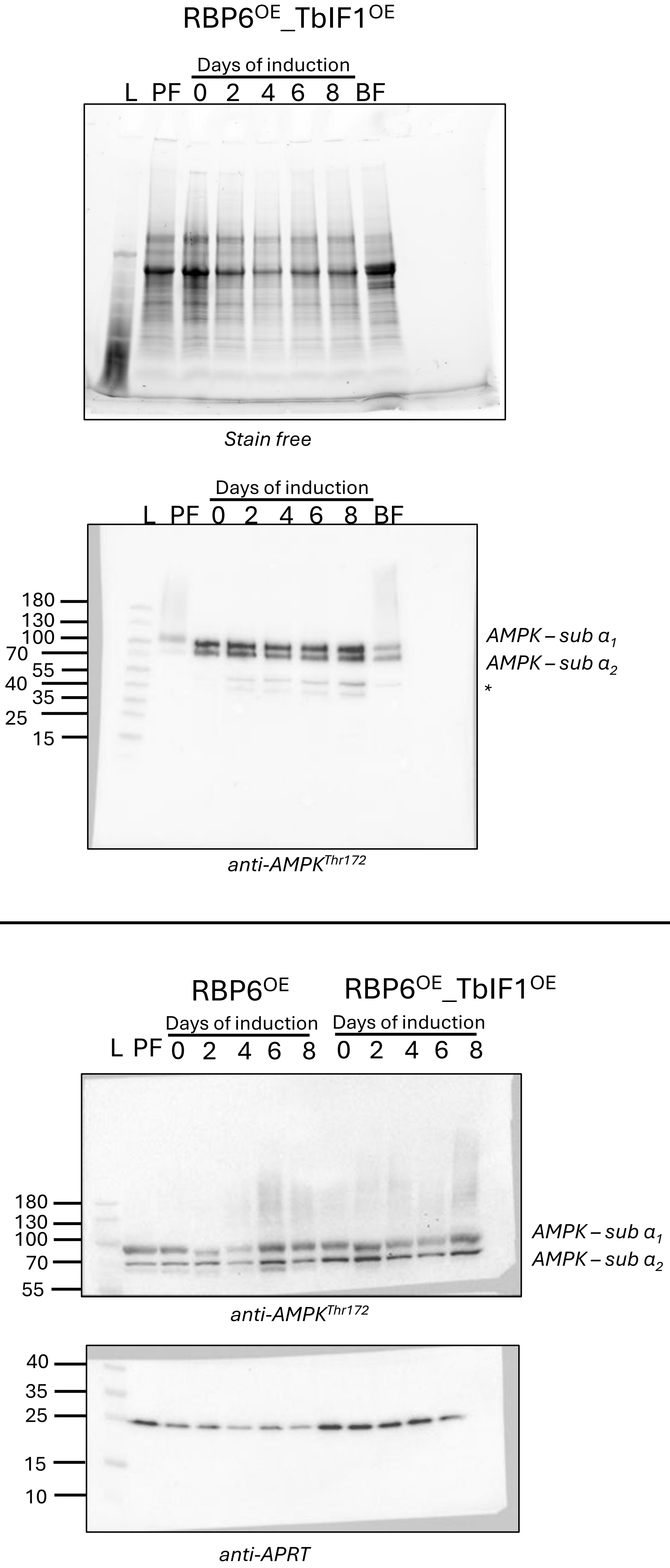

* - nonspecific band
L – ladder
PF – procyclic form wt
BF – bloodstream form wt

Figure 6C

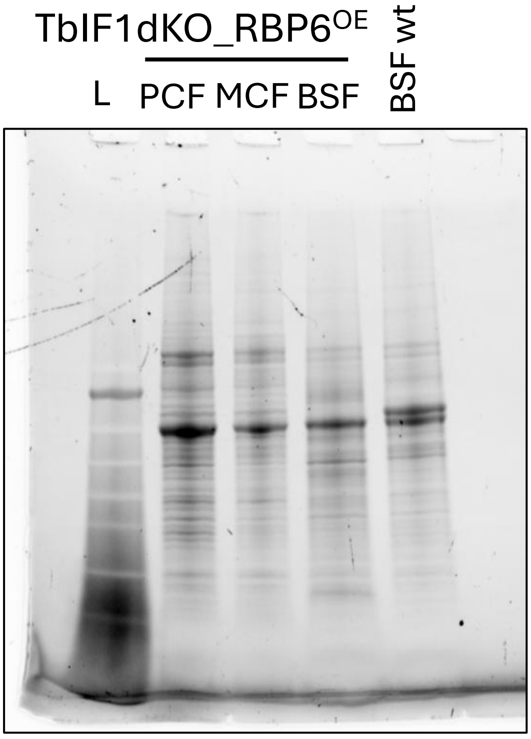

Stain free

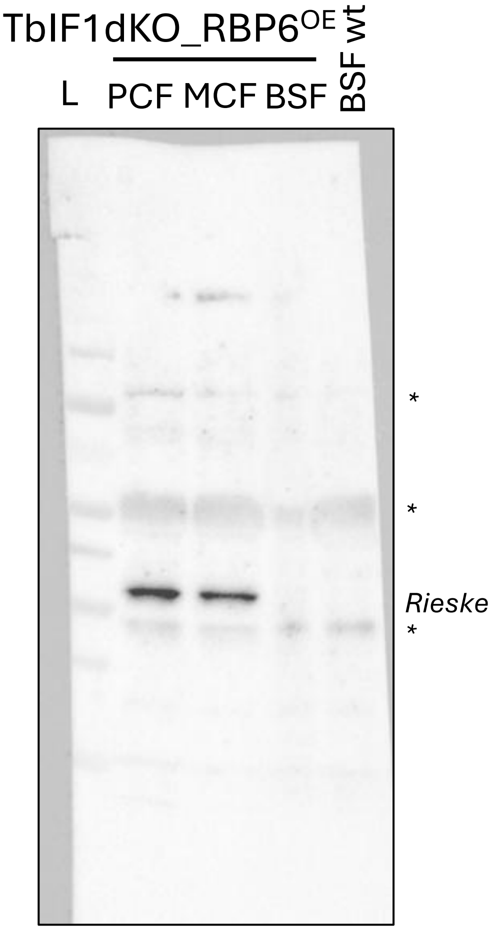

Anti-Rieske

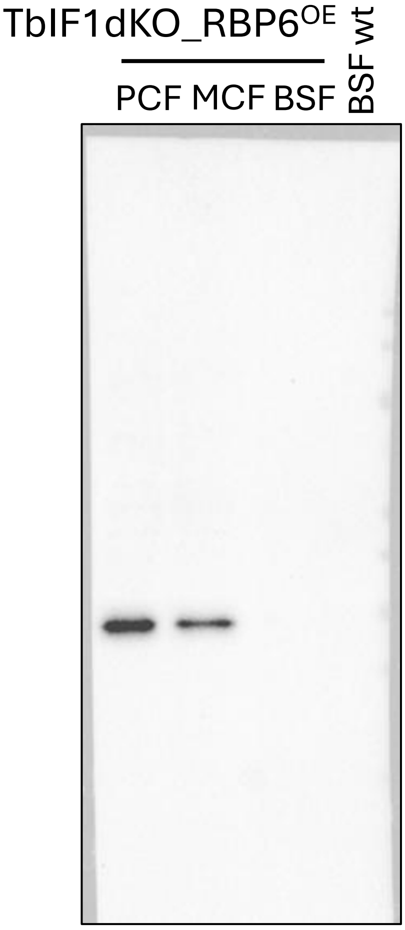

Anti-trCOIV

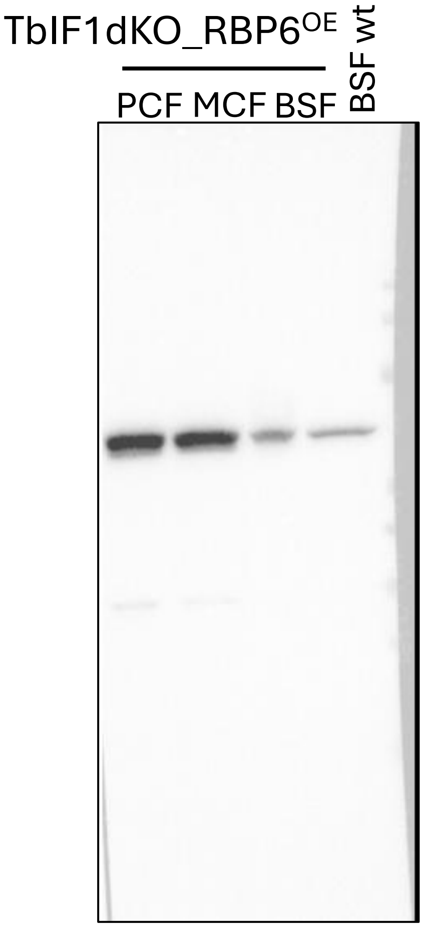

anti-mtHSP70

* - nonspecific band
L – ladder
PCF – procyclic form
MSF -metacyclic form
BSF – bloodstream form wt

Figure 6C

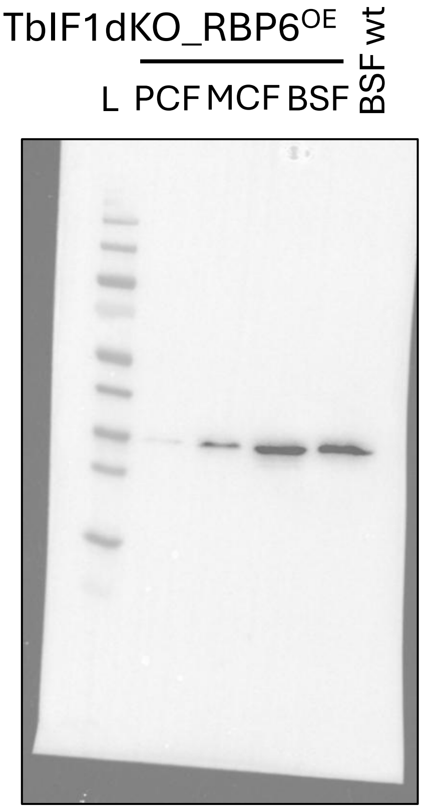

Anti-AOX

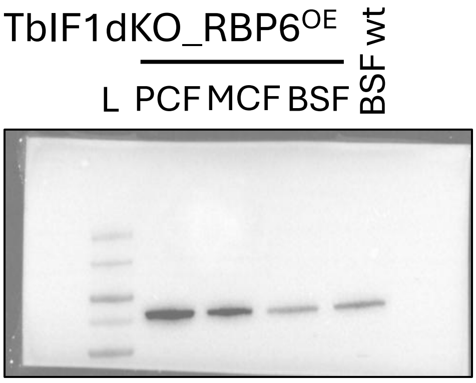

Anti-mtHSP70

* - nonspecific band
L – ladder
PCF – procyclic form
MSF -metacyclic form
BSF – bloodstream form wt

Supplementary Figure S3

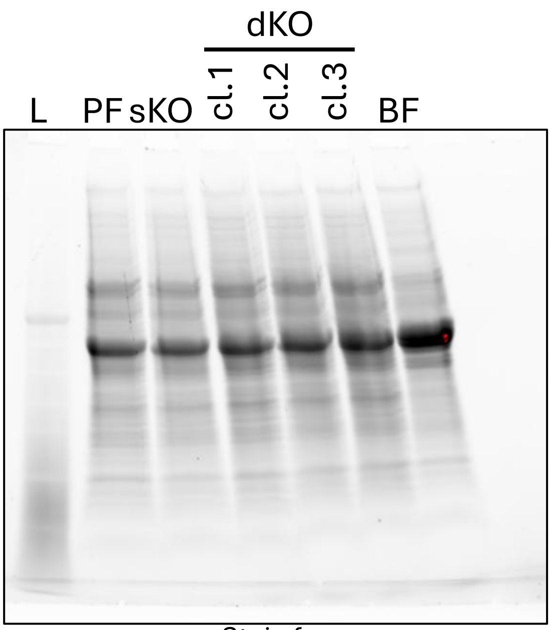

Stain free

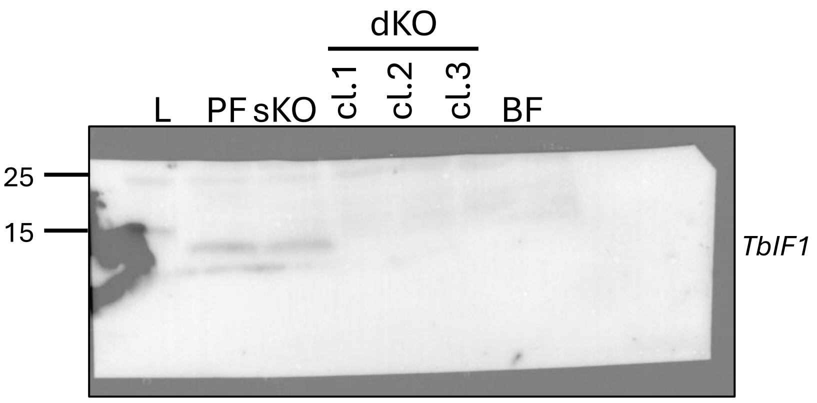

anti-TbIF1

Supplementary Figure S3

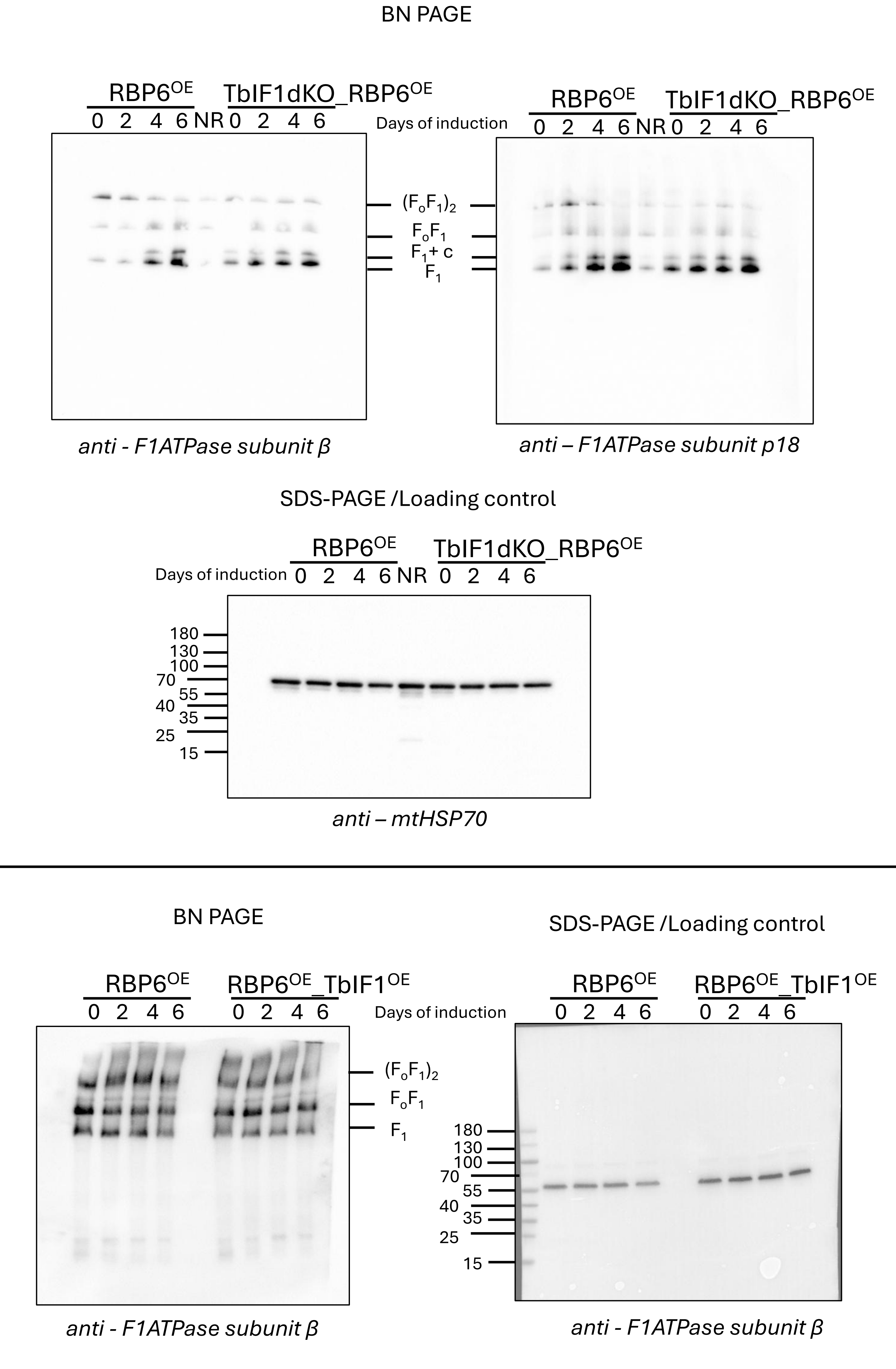

* - nonspecific band

L – ladder

NR – non-relevant procyclic cells sample

Supplementary Figure S1

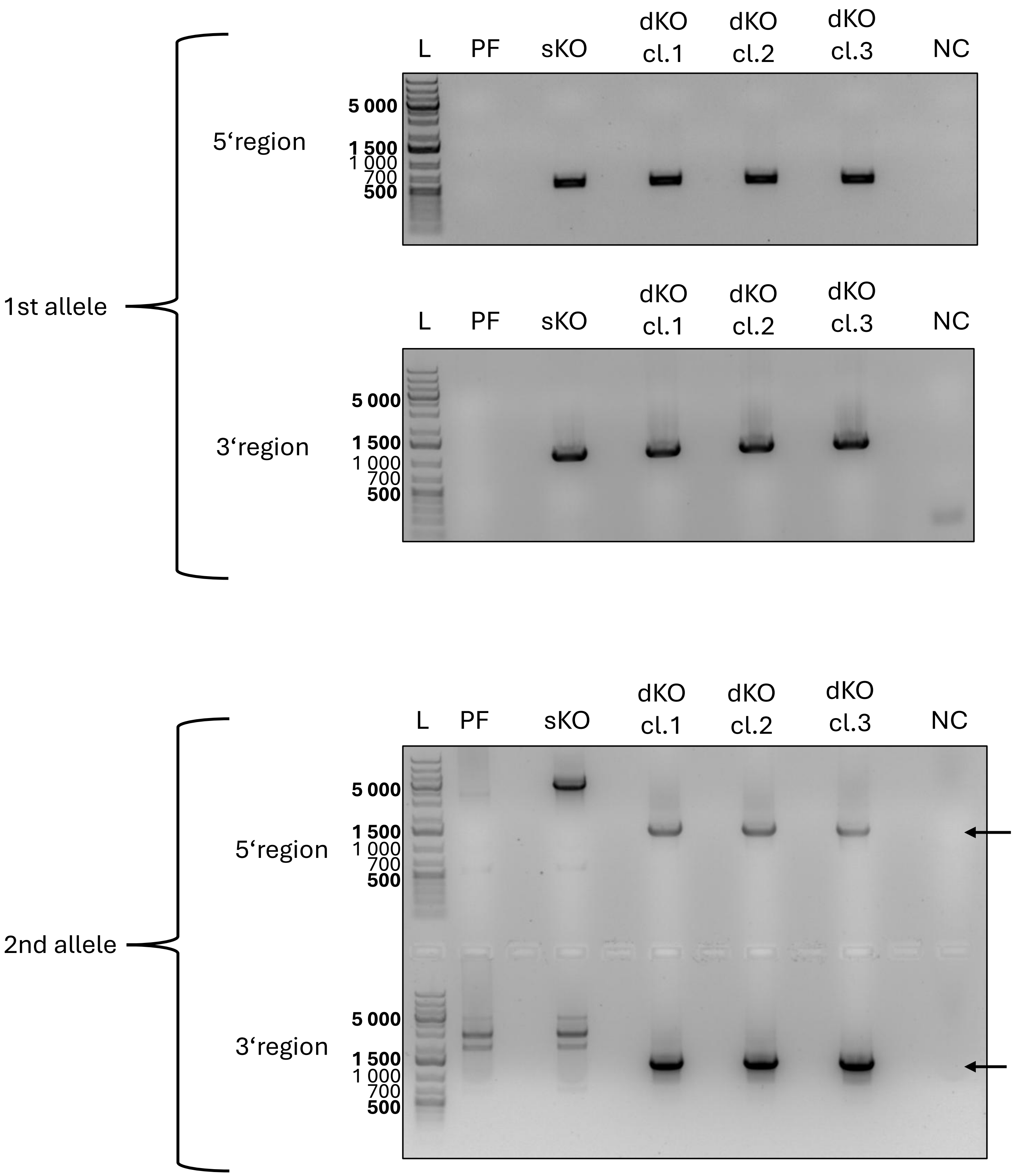

L – ladder
NC – negative control
PF – procyclic form wt
dKO cl. 1-3 – dKO cell line
