# Supplementary material for: Reversal of ATP synthase is a key attribute accompanying cellular differentiation of Trypanosoma brucei insect forms
Source: Commun Biol. 2026 Mar 27;9:680. doi: 10.1038/s42003-026-09933-z (PMC13187271; doi:10.1038/s42003-026-09933-z)
Supplement: Supplementary file 2 — Description of Additional Supplementary Files [file 42003_2026_9933_MOESM2_ESM.docx]

**Description of Additional Supplementary Files**

**File name:** Supplementary Data 1

**Description:** The source data for all charts/graphs, description of T. brucei strains and list of used oligonucleotides.
